# 25 Effect of Dry Hydrogen Peroxide on Candida auris in ICU Settings

**DOI:** 10.1017/ash.2026.10470

**Published:** 2026-06-23

**Authors:** Fraser Mackay, Alexander Pyden, Calvin Brown, Jiwon Chung, Caroline Schissel, Meaghan Karolides, Andrew Goldsmith

**Affiliations:** 1 Lahey Hospital and Medical Center; 2 Lahey Hospital & Medical Center

## Abstract

**Background:** Early identification of severe sepsis or septic shock in the emergency department (ED) is challenging; patients triaged to the waiting room commonly encounter hours of delay before provider evaluation. Although vital signs and laboratory testing may be obtained at check-in, patients may deteriorate during this waiting period, and so there is a critical need for indicators of occult disease. Common inflammatory biomarkers such as C-reactive protein (CRP) are sensitive but poorly specific in undifferentiated ED populations and offer limited value for early risk stratification. We evaluated the diagnostic performance of an FDA-cleared host-response RNA biomarker assay (SeptiCyte RAPID) for identifying severe sepsis or septic shock prior to clinician evaluation. **Method:** As part of a multi-phase validation study, we conducted a prospective observational cohort study at a tertiary-care ED. During predefined monthly windows we consecutively enrolled adult patients (≥18 years) if they: were triaged for suspected infection, had ≥2 systemic inflammatory response syndrome (SIRS) criteria, blood drawn at triage, and were initially placed in the waiting room or non-treatment areas. The SeptiCyte assay was performed as an add-on study using blood already collected, with clinicians blinded to results. Severe sepsis or septic shock was adjudicated retrospectively using standardized SEP-1 abstraction criteria published by the Centers for Medicare and Medicaid Services (CMS). Diagnostic performance was assessed using sensitivity, specificity, area under the receiver operating characteristic curve (AUC), and likelihood ratios. Likelihood ratios were contextualized against published CRP performance in undifferentiated ED populations. **Result:** We enrolled 101 patients in this phase, and at a prespecified higher-risk threshold, SeptiCyte demonstrated a sensitivity of approximately 0.59 and specificity of 0.77, with an AUC of 0.69 and a positive likelihood ratio of 2.6. In comparison, published CRP performance in similar undifferentiated ED populations is characterized by AUCs ranging from approximately 0.60–0.68 and positive likelihood ratios ≤2.0, reflecting lower specificity. **Conclusion:** In triaged ED patients with suspected infection awaiting emergency provider evaluation, a host-response RNA biomarker demonstrated moderate discrimination for subsequent severe sepsis or septic shock with operating characteristics favoring specificity and rule-in capability. Compared with published CRP performance, the biomarker appears better suited for early risk stratification and flag earlier clinician evaluation rather than broad inflammatory screening in ED waiting-room populations.

